# Novel Biomarkers of Arterial and Venous Ischemia in Microvascular Flaps

**DOI:** 10.1371/journal.pone.0071628

**Published:** 2013-08-14

**Authors:** Gerard K. Nguyen, Brian H. Hwang, Yiqiang Zhang, John F. W. Monahan, Gabrielle B. Davis, Yong Suk Lee, Neli P. Ragina, Charles Wang, Zhao Y. Zhou, Young Kwon Hong, Ryan M. Spivak, Alex K. Wong

**Affiliations:** 1 Division of Plastic & Reconstructive Surgery, Department of Surgery, Keck School of Medicine, University of Southern California, Los Angeles, California, United States of America; 2 City of Hope, Functional Genomics Core, Duarte, California, United States of America; 3 Division of Cardiology, Department of Medicine, University of Washington, Seattle, Washington, United States of America; 4 Division of Colorectal Surgery, Department of Surgery, Keck School of Medicine, University of Southern California, Los Angeles, California, United States of America; 5 Department of Surgery and Biochemistry & Molecular Biology, Keck School of Medicine, University of Southern California, Los Angeles, California, United States of America; Harvard Medical School, United States of America

## Abstract

The field of reconstructive microsurgery is experiencing tremendous growth, as evidenced by recent advances in face and hand transplantation, lower limb salvage after trauma, and breast reconstruction. Common to all of these procedures is the creation of a nutrient vascular supply by microsurgical anastomosis between a single artery and vein. Complications related to occluded arterial inflow and obstructed venous outflow are not uncommon, and can result in irreversible tissue injury, necrosis, and flap loss. At times, these complications are challenging to clinically determine. Since early intervention with return to the operating room to re-establish arterial inflow or venous outflow is key to flap salvage, the accurate diagnosis of early stage complications is essential. To date, there are no biochemical markers or serum assays that can predict these complications. In this study, we utilized a rat model of flap ischemia in order to identify the transcriptional signatures of venous congestion and arterial ischemia. We found that the critical ischemia time for the superficial inferior epigastric fasciocutaneus flap was four hours and therefore performed detailed analyses at this time point. Histolgical analysis confirmed significant differences between arterial and venous ischemia. The transcriptome of ischemic, congested, and control flap tissues was deciphered by performing Affymetrix microarray analysis and verified by qRT-PCR. Principal component analysis revealed that arterial ischemia and venous congestion were characterized by distinct transcriptomes. Arterial ischemia and venous congestion was characterized by 408 and 1536>2-fold differentially expressed genes, respectively. qRT-PCR was used to identify five candidate genes Prol1, Muc1, Fcnb, Il1b, and Vcsa1 to serve as biomarkers for flap failure in both arterial ischemia and venous congestion. Our data suggests that Prol1 and Vcsa1 may be specific indicators of venous congestion and allow clinicians to both diagnose and successfully treat microvascular complications before irreversible tissue damage and flap loss occurs.

## Introduction

Advances in reconstructive microsurgery have created a paradigm shift in the restoration of form and function, allowing for the dynamic repair of devastating injuries, severely diseased tissue, and previously unreconstructible wounds [1]–[18]. Composite tissue allotransplantation of the face and upper extremity is now a reality, with over seventy procedures performed to date and an increasing amount of clinical centers offering these operations to their patients. Transplantation of composite constructs of thorax, abdominal wall, pelvis, and urogenital systems is inevitable and will offer hope to those patients with the most severe pathology. Lower extremity free tissue transfer is commonplace at most tertiary centers for limb salvage in cases that would otherwise proceed to amputation. Breast reconstruction restores form and psychological well-being to women with breast cancer. Creation of neural-integrated bioprosthetic devices, interfaces between human tissue and robotics, has created an avenue of hope for upper extremity amputees. Finally, advances in tissue engineering and de-novo organogenesis rely on bioreactors, mechanical pumps that supply nutrient flow for growth and elimination of toxic metabolites to allow for creation of tissues and organs. Essential to each of these procedures is the creation of a nutrient vascular supply, via surgically fashioned microscopic anastamoses between single arteries and veins, ranging in size from 0.5 to 3.0 millimeters. Surgical complications related to occluded arterial inflow and obstructed venous outflow can result in irreversible injury to tissues and may result in tissue compromise and necrosis. Oftentimes, these complications are difficult to clinically determine [19]. As early intervention with return to the operating room to re-establish inflow or outflow is the essential step in flap salvage, the accurate diagnosis of early stage vascular complications is key [20]. To date, however, there are no biochemical markers or serum assays that can predict these complications. In this animal study of free tissue transfer failure, we have created a model for venous congestion and arterial ischemia in order to identify novel biomarkers for early prediction of flap complications. While tissue ischemia due to decreased or absent arterial flow is a well-studied physiologic phenomenon, the process of venous congestion is not as well-characterized. The pathophysiology of arterial occlusion results in an inadequate oxygen supply, and simultaneous deficit in clearance of toxic metabolites to affected tissues. This results in the accumulation of reactive oxygen species (ROS), an influx of inflammatory cells including neutrophils, macrophages, and a progressive release of cytokines in a cycle of inflammation that ultimately leads to tissue necrosis [21]–[24]. ROS are the major causative factors that link the biochemical pathways between persistent ischemia and tissue necrosis, causing microcirculatory damage that results in irreversible deterioration and injury [25], [26]. In contrast, venous congestion has been less frequently studied, but characterized mainly in microvascular flaps, as well as the gastrointestinal tract [27], brain [28]. In venous congestion, arterial flow persists, causing increased intravascular pressure and subsequent hemorrhage of the microvasculature into the extra-vascular space [29]–[31]. The increased extra-vascular pressure causes external compression and collapse of the vessels. The edema that forms in the interstitial tissue acts as a barrier to the diffusion of oxygen, further contributing to tissue damage [32], [33].

Because of the dependence of a single artery for inflow and a single vein for outflow, microvascular flaps represent a compelling model to compare the physiological differences between arterial ischemia and venous congestion. Although brief episodes of ischemia and reperfusion are tolerated, there comes a critical “point of no return” where flaps cannot be salvaged following prolonged periods of ischemia [34]. Moreover, complications involving venous congestion after microvascular free tissue transfer are more common due to the inherent low flow state of tissue as well increased compliance of the vein relative to the artery that is less resistant to compression. In addition venous congestion is hypothesized to be more clinically detrimental to flaps than arterial ischemia [35]–[39], suggesting that these two biological processes are distinct in their pathophysiology.

To better understand the transcriptional changes that occur following arterial ischemia and venous congestion, the goal of this study was to identify novel biomarkers in a high fidelity rat model of microvascular free flap failure. It has been established that changes in gene expression occur rapidly following arterial ischemia. Thus, we set out to identify these rapid changes in gene expression in order to better distinguish arterial ischemia from venous congestion. Only a limited number of studies have compared transcriptional changes in arterial ischemia and venous congestion. Zhang *et al.* used a skin flap model and found that tumor necrosis factor (TNF-alpha) was significantly up-regulated in arterial ischemia, whereas monocyte chemoattractant protein-1 (MCP-1) was associated with venous congestion [40]. Mithani *et al.* used a rat flap model to identify genes related to flap failure, but only studied venous congestion [41]. The goal of this study is to perform a thorough comparative analysis of arterial ischemia and venous congestion using gene expression microarray analysis [42], [43]. We hypothesize that early changes in gene expression in response to arterial ischemia and venous congestion occur within minutes of inflow and outflow obstruction. An enhanced understanding of the gene expression profiles between arterial ischemia and venous congestion will lay the groundwork for further mechanistic studies, and ultimately a more nuanced understanding of their pathophysiology. Finally, the development of biomarkers specific to arterial ischemia or venous congestion would represent a clinical translational application of this technology for early clinical diagnosis of flap failure by bedside serum and blood level assay. Early diagnosis of flap-threatening arterial ischemia and venous congestion would represent a paradigm shift in our ability to treat these rapidly progressive processes. Early and rapid diagnosis with a serum biomarker could translate to higher rates of free flap salvage; this would represent a significant advance in the safety, quality, and effectiveness of care of patients requiring free tissue transfer by microvascular surgical techniques.

## Materials and Methods

### Animals

Animal experiments were conducted in compliance with the protocol approved by The University of Southern California Institutional Animal Care and Use Committee (IACUC Protocol #11463). Male Sprague Dawley rats weighing 300–325 g were induced and maintained on 2% isoflurane by use of an anesthesia system (Molecular Imaging Products Company, Bend, OR). All operative procedures were performed using standard aseptic technique [44]. The animals were housed in individual cages and fed standard rat chow and water ad libitum during their post-operative recovery.

### Operative Procedure

**Figure 1:**
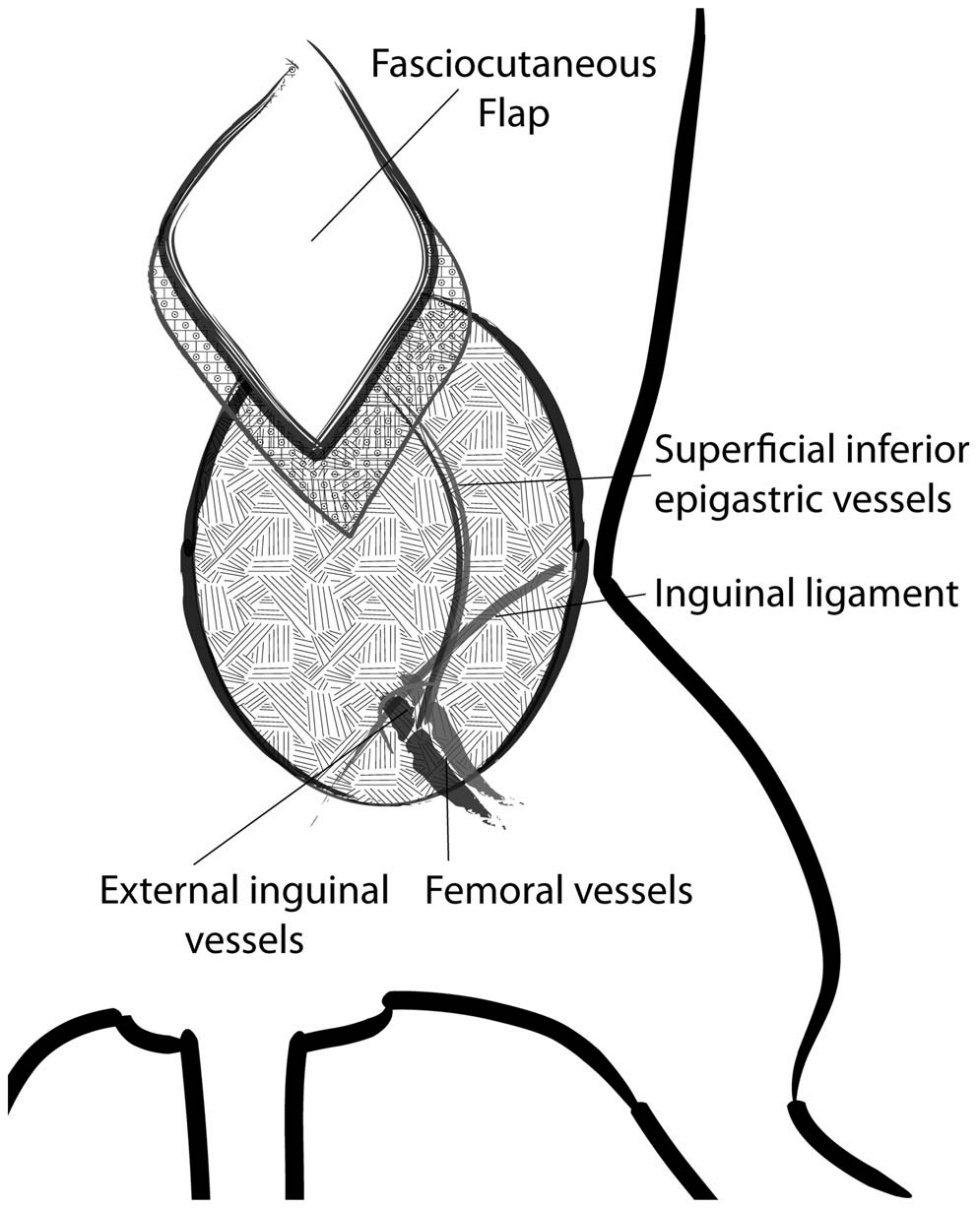
Diagram of operative procedure, phenotype analysis. A) Diagram of lower left inguinal anatomic landmarks of flap with labeling of superficial inferior epigastric artery, vein, and inguinal ligament.

A modified version of a well-described technique of flap elevation based off the superficial inferior epigastric (SIE) vessels was performed [35]. The abdominal and groin regions were shaved and the hair over these regions was removed. The area was then prepped with the antiseptic/antimicrobial povidone-iodine scrub solution. A 2×2 cm, rhomboid-shaped flap was marked in the area overlying the territory of the SIE artery and vein (thus comprising the SIEA flap). The flap was raised with careful dissection to the SIE artery and vein (Figure 1). With good exposure and visualization of the SIE artery and vein, the adventitia surrounding both structures was gently cleaned and removed. The SIE nerve was located and ligated. A microvascular clamp (S&T AG, Neuhausen, Switzerland) was then used to clamp either the SIE artery or vein for varying timepoints for the experimental arm of the study. After the experimental arm of the study was completed, the SIEA flap was inset into its original position in the rat, and the incisions closed.

### Phenotype Analysis

Temporal phenotypic changes between treatment and control groups were analyzed grossly. In a set of animals, the SIEA flap was created as described above and venous congestion was induced for varying time periods (1, 2, 2.5, 3, 3.5, and 4 hours) after which the vein was unclamped to allow for reperfusion. Similarly, arterial ischemia was induced for the same respective time periods. The flap was then inset as previously described, and gross phenotypic changes were observed until post-operative day 6. Imaging to determine flap survival was captured using a digital camera (Canon PowerShot S90, Tokyo, Japan). The area of flap survival was determined using ImageJ (U.S. National Institutes of Health, Bethesda, MD). Phenotypic changes were also evaluated using histology. Flap tissue at the end of 4 hours of arterial ischemia or venous congestion was harvested, fixed in 10% buffered formalin, embedded in paraffin, and sent for standard hematoxolyin and eosin (H&E) staining.

### Tissue Collection and RNA Extraction

Following the period of ischemia, the animals were re-anesthetized for the removal of the clamp. The microvascular flaps undergoing four hours of arterial ischemia (n = 5) and venous congestion (n = 5), and a control non-ischemic group (n = 5) were harvested subsequently and stored in a freezer at −80°C. Total RNA was extracted using the RNeasy kit (Qiagen, Hilden, Germany) following the manufacturer’s protocol, and quality checked using Agilent BioAnalyzer Nano 6000 chip (Agilent, Santa Clara, CA).

### Microarray Hybridization

For the microarray study, 3′IVT Expression kit (Affymetrix, Santa Clara, CA) was used to synthesize first-strand cDNA from each total RNA sample with the use of T7-ologo (dT) primer, and second–strand using DNA polymerase while simultaneously removed RNA; T7 in vitro transcription (IVT) technology was employed in the kit to synthesize amplified RNA (aRNA) that was simultaneously labeled with biotin. Labeled aRNA was purified by magnetic beads per manufacture’s protocol, fragmented, and was subsequently (10 µg) hybridized on rat genome 230 2.0 GeneChip (Affymetrix) to analyze the expression profile of 31,042 probe sets. After washing, the arrays were stained with streptavidin-phycoerythrin, and the signal amplified by biotinylated anti-streptavidin (Vector Laboratories, Inc., Burlingame, CA), and then scanned on an Affymetrix GCS 3000 7 G scanner. The intensity for each feature of the array was captured using Affymetrix GeneChip Command Console (AGCC), according to the standard Affymetrix procedures. Gene expression values were extracted using Affymetrix Gene Expression Console.

### Generation of Differentially Expressed Gene Lists

Data from the Affymetrix Gene Expression Console were imported into Partek Genomics Suites 6.5 (Partek, St. Louis, MO) to generate results from principal component analysis (PCA), hierarchical clustering analysis, and lists of differentially expressed genes (DEG). Lists of DEG were generated with the following parameters: absolute value fold-change ≥2 compared to control, with false discovery rate (FDR)-adjusted *P* value ≤0.05. Microarray data were deposited in the National Center for Biotechnology Information’s Gene Expression Omnibus (GEO) database with accession number GSE35270 (http://www.ncbi.nlm.nih.gov/geo/query/acc.cgi?acc=GSE35270).

Lists of DEG were characterized by Gene Ontology (GO) attributes with FuncAssociate 2.0 (llama.mshri.on.ca/funcassociate/) [45]. Sets were also analyzed by the Ingenuity Pathway Analysis tool (IPA 8.7, Ingenuity Systems, San Francisco, CA) to visualize changes in gene expression and rank by score top associated network functions, biological functions, and canonical pathways.

### Validation of Gene Expression by Quantitative Real-time PCR (qRT-PCR)

For qRT-PCR validation, arterial (n = 5), venous (n = 7), and control (n = 5) flaps were analyzed at 4 hours of occlusion. Additional flaps undergoing venous congestion at 1 (n = 3) and 3 (n = 3) hours were also used for qRT-PCR analysis. On the basis of the microarray data, we selected to validate by qRT-PCR the expression of 5 target genes *Fcnb*, *Il1b*, *Muc1*, *Prol1* (*Muc10*), and *Vcsa1*, and a reference gene *Actb* (beta actin) using TaqMan Universal Mix II kit (Applied Biosystems, Carlsbad, CA). The qRT-PCR assays and pre-designed and verified primers were purchased from Life Technology (Carlsbad, CA) (Table S1). 100 ng of total RNA extracted from tissues (arterial ischemia, venous congestion, or control) were reversed-transcribed to obtain cDNA using a SuperScript VILO kit (Invitrogen, Carlsbad, CA, USA). Duplex qRT- PCR reactions detecting *Actb* and each target gene were performed using standard thermal cycling protocol on ABI ViiA 7 RT-PCR system (Applied Biosystems). The thermal cycle consisted of 95°C for 10 min followed by 40 two-step cycles of 95°C for 15 s and 60°C for 1 min. Relative quantification using Ct values and the comparative 2^−ΔΔCt^ method was used to evaluate the fold change of genes in arterial ischemia and venous congestion groups as compared to control samples [46]. The expression levels of *Actb* were used for gene expression normalization.

### Statistical Analysis

Results are presented as means ± SE. Data were analyzed using Prism (GraphPad Software Inc., San Diego, CA). Differences between means of two groups were analyzed by a Student’s t-test. Comparison between treatment and control groups for the microarray study was conducted with 1-way ANOVA [47]. Logarithmic values of the gene expression qRT-PCR data were used for determination of significance [48]. *P*-values of <0.05 were considered statically significant.

## Results

### Determination of Flap Survival in Arterial Ischemia and Venous Congestion

**Figure 2:**
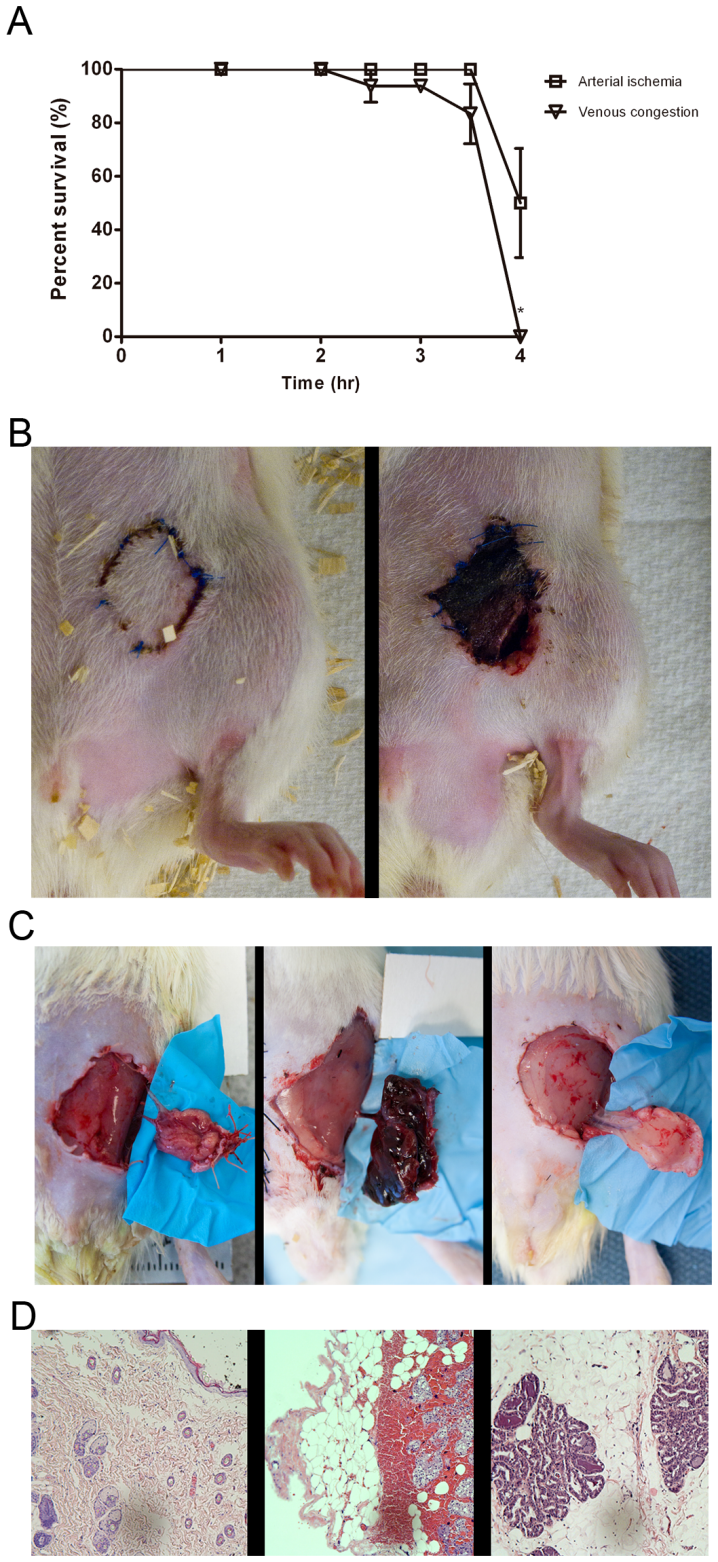
Phenotype analysis, temporal profile of flap survival following vessel occlusion. A) Bar graph of percent flap survival of ischemia-reperfusion vs. time on postoperative day 6 of arterial ischemia and venous congestion. Data are expressed as means ± SE and analyzed by unpaired *t*-test. *P<0.05. B) Gross photos of flaps at 3.5 vs. 4 hours of venous congestion C) From left to right, gross photos of flaps subjected to 4 hours of arterial ischemia and venous congestion compared to control D) Histology showing flaps subjected to 4 hours of venous congestion showing extravasation of blood, while control specimen and arterial ischemia lack such findings.

Characterization of flap survival was performed following ischemic stress. The duration of ischemia that results in irreversible damage to the flap defines the time from which to assess the sequential molecular events that lead to flap necrosis. To determine this critical time of ischemia, an ischemia-reperfusion study was performed. Overwhelmingly, flaps were able to recover fully after 3 hours of venous or arterial occlusion (Figure 2A). However, 4 hours of venous congestion resulted in significantly lower rates of flap survival at 17.25%, as compared to 80% flap survival (*P* = 0.036) upon 3.5 hours of venous congestion (Figure 2B). In contrast, no significant difference in flap survival was observed for arterial ischemia at 4 hours compared to 3.5 hours (*P* = 0.313). Flaps after 4 hours of venous congestion showed reduced survival compared to arterial ischemia, however the difference did not reach statistical significance (*P = *0.292).

### Gross and Histologic Findings in Venous Congestion and Arterial Ischemia

Given that the 4-hour timepoint appeared to be critical for the survival of ischemic flaps, we sought to characterize the model specifically at this time with gross examination and histology of the flap tissue. After 4 hours of venous congestion, the veins were completely thrombosed and flaps appeared to be grossly edematous, cyanotic, and warm compared to the surrounding non-ischemic tissue. The arterial flap, however, appeared to be of constant size, pale, and cool (Figure 2C). Histologic analysis revealed that flaps which were subjected to 4 hours of venous congestion showed extravasation of red blood cells, while those subjected to arterial ischemia and control specimens lacked such findings (Figure 2D). These data demonstrate that arterial ischemia and venous congestion present profound differences in gross phenotype, histology, and flap survival.

### Global Transcriptome Profiles in Ischemic Flaps

**Figure 3:**
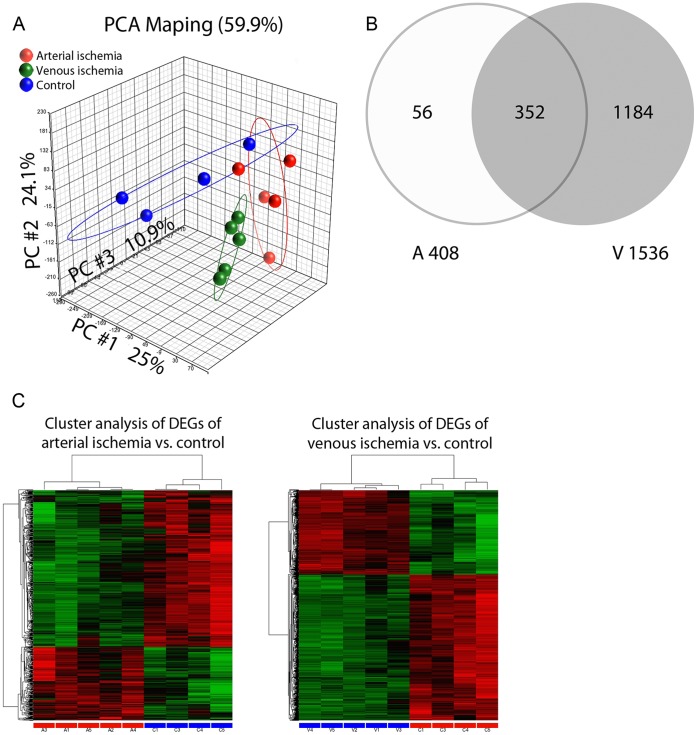
Global transcriptome profiles in ischemic flaps. A) PCA diagram B) Venn diagram of differentially expressed genes upon 4 hours of arterial ischemia and venous congestion C) Hierarchical cluster analysis of the differentially expressed genes in rats following 4 hours of arterial ischemia and venous congestion.

Because 4 hours of ischemia in flaps show a substantial difference in survival rate, we proposed that this characteristic pathology is accompanied with changes in global gene expression. As shown in Figure 3A, a principal component analysis (PCA) revealed clear segregation between groups. The first, second, and third components represent 25%, 24.1%, and 10.9% of the transcriptome respectively. The cumulative proportion of 59.9% indicates that the three components represent most of the expression pattern. Differentially expressed gene (DEG) lists (http://www.ncbi.nlm.nih.gov/geo/query/acc.cgi?acc=GSE35270) for arterial ischemia and venous congestion were generated as outlined in the methods section. Of the venous congestion and arterial ischemia lists, there were 352 overlapping DEGs, representing 86.2% of the DEGs from arterial ischemia and 22.9% of the DEGS from the venous congestion lists (Figure 3B).

### Differential Gene Expression in Flaps Undergoing Arterial Ischemia

In flaps undergoing arterial ischemia, the DEG list comprised 408 genes. Of these, 95 genes were up-regulated, and 313 genes were down-regulated. Hierarchical clustering analysis demonstrates a clear separation between control and treatment groups, which supports the validity of the microarray analysis. In addition, the samples sent for analysis from the control and treatment groups presented with similar genomic profiles (Figure 3C). To understand vascular-specific pathology of microvascular flap gene expression, we identified differences in biological processes that arterial ischemia induces compared to control using gene ontology (GO) analysis. GO analysis sorts DEGs of arterial ischemia by gene product functions, or attributes. A total of 203 GO attributes were identified with adjusted *P* values of <0.01 between arterial ischemia and control. Not surprisingly, top attributes of arterial ischemia samples related to immune responses (Table 1). In addition to gene functions, we identified gene pathways of the DEGs for arterial ischemia using Ingenuity Pathway Analysis (IPA) software (Table 2). The top three IPA associated network functions and top two biological functions were related to inflammatory responses. Other top biological functions included immunological disease and immune cell trafficking. Of note, a top canonical pathway listed was production of nitric oxide and reactive oxygen species in macrophages, which supports previous knowledge of pathways involved in arterial ischemia [21]. In conclusion, IPA suggests an increase in inflammatory and immunological activity in flaps undergoing arterial ischemia compared to control.

**Table 1:** Top GO classes of differentially expressed genes.

*P*-value	Attributed name
**Arterial ischemia vs. control DEG**	
**4.66E-19**	immune system process
**4.88E-18**	defense response
**1.62E-16**	immune response-regulating signaling pathway
**3.37E-16**	immune response-activating signal transduction
**4.90E-16**	activation of immune response
**1.53E-15**	immune effector process
**4.00E-15**	regulation of immune response
**8.15E-15**	positive regulation of immune response
**8.56E-15**	positive regulation of response to stimulus
**5.52E-14**	immune response-regulating cell surface receptor signaling pathway
**8.36E-14**	response to other organism
**1.16E-13**	regulation of response to stimulus
**Venous congestion vs. control DEG**	
**1.50E-22**	regulation of multicellular organismal process
**1.81E-22**	immune system process
**5.88E-19**	positive regulation of immune system process
**1.17E-18**	regulation of localization
**4.05E-18**	cellular response to chemical stimulus
**2.58E-17**	response to organic substance
**7.36E-16**	defense response
**1.21E-15**	regulation of immune response
**1.91E-15**	regulation of immune system process
**2.08E-15**	regulation of cytokine production
**4.77E-15**	response to wounding
**1.29E-14**	positive regulation of cell activation
**1.51E-14**	positive regulation of response to stimulus
**2.69E-14**	response to stress
**Overlapping DEG**	
**1.25E-20**	immune system process
**2.07E-19**	defense response
**3.05E-17**	immune effector process
**6.86E-17**	immune response-regulating signaling pathway
**1.37E-16**	activation of immune response
**1.70E-16**	immune response-activating signal transduction
**1.95E-16**	regulation of immune response
**8.51E-16**	response to other organism
**1.09E-15**	positive regulation of immune response
**3.52E-15**	immune response-regulating cell surface receptor signaling pathway
**1.59E-14**	positive regulation of immune system process
**1.62E-14**	immune response-activating cell surface receptor signaling pathway
**1.86E-14**	regulation of multicellular organismal process

**Table 2:** Ingenuity Pathway Analysis results for differentially expressed genes in rat composite microvascular flaps undergoing 4 hours of arterial ischemia and venous congestion compared to control flaps.

	Score	*P*-value	Genes, *n*
*Arterial Ischemia*			
Top Associated Network Functions			
Infectious Disease, antigen presentation, inflammatory response	33		
Cellular compromise, cellular function and maintenance, inflammatory response	30		
Cellular function and maintenance, cell-to-cell signaling and interaction, inflammatory response	28		
Cellular movement, hematological system development and function, cell-to-cell signaling and interaction	28		
Cellular movement, hematological system development and function, immune cell trafficking	25		
Top biological functions			
Inflammatory response		1.02E-21–1.48×10^−04^	108
Inflammatory disease		8.11E-16–1.03×10^−04^	122
Immunological disease		9.57E-14–1.48×10^−04^	112
Organismal injury and abnormalities		2.37E-12–1.48×10^−04^	53
Respiratory disease		4.10E-12–1.48×10^−04^	49
Cellular development		5.84E-24–1.52×10^−04^	112
Cellular movement		9.35E-18–1.39×10^−04^	83
Cellular function and maintenance		3.62E-15–1.52×10^−04^	87
Cell death		5.26E-15–1.48×10^−04^	130
Cell-to-cell signaling and interaction		6.45E-15–1.48×10^−04^	98
Hematological system development		8.74E-20–1.61×10^−04^	120
Tissue morphology		8.74E-20–7.04×10^−05^	74
Immune cell trafficking		9.35E-18–1.61×10^−04^	79
Hematopoiesis		2.40E-17–1.52×10^−04^	78
Cell-mediated Immune Response		8.18E-14–1.52×10^−04^	54
Top Canonical Pathways			
Colorectal cancer metastasis signaling		9.65×10^−7^	
Type I diabetes mellitus signaling		1.35×10^−6^	
Production of nitric oxide and reactive oxygen species in macrophages		1.85×10^−6^	
Role of macrophages, fibroblasts and endothelial cells in rheumatoid arthritis		2.22×10^−6^	
Role of pattern recognition receptors in recognition of bacteria and viruses		2.57×10^−6^	
*Venous congestion*			
Top associated network functions			
Renal and urological disease, cell death, embryonic development	41		
Nutritional disease, immunological disease, gastrointestinal disease	32		
Post-translational modification, cell death, nervous system development and function	32		
Cell signaling, cardiovascular system development and function, connective tissue disorders	31		
Genetic disorder, immunological disease, cardiovascular disease	30		
Top biological functions			
Inflammatory response		1.07E-28–2.44×10^−07^	262
Cancer		4.81E-27–1.72×10^−07^	423
Inflammatory disease		4.58E-25–2.59×10^−07^	384
Immunological disease		7.44E-23–2.30×10^−07^	326
Hematological disease		2.23E-17–3.17×10^−07^	250
Cellular movement		1.94E-34–3.17×10^−07^	285
Cellular growth and proliferation		6.15E-34–2.49×10^−07^	396
Cellular development		7.63E-27–2.47×10^−07^	352
Cell death		3.31E-23–2.53×10^−07^	371
Cell-to-cell signaling and interaction		1.05E-22–2.01×10^−07^	268
Hematological system development and function		3.36E-33–2.30×10^−07^	291
Immune cell trafficking		3.36E-33–2.27×10^−07^	196
Tissue morphology		4.73E-24–2.30×10^−07^	186
Organismal survival		9.46E-23–2.05×10^−08^	201
Tissue development		1.05E-22–2.13×10^−07^	198
Top Canonical Pathways			
Type I diabetes mellitus signaling		1.05×10^−10^	
Production of nitric oxide and reactive oxygen species in macrophages		1.28×10^−7^	
Hepatic fibrosis/hepatic stellate cell activation		1.90×10^−7^	
IL-10 signaling		3.96×10^−7^	
T helper cell differentiation		5.02×10^−7^	

### Differential Gene Expression in Flaps Undergoing Venous Congestion

The DEG list of flaps subjected to venous congestion comprised 1536 genes. Of these, 606 genes were up-regulated and 930 genes were down-regulated. Hierarchical clustering analysis demonstrates a clear separation between control and treatment groups. Samples from within the control and treatment groups predictably presented with similar profiles (Figure 3C). GO analysis sorts DEGs of venous congestion by gene product functions, or attributes. A total of 258 GO attributes were identified with adjusted *P* values of <0.01 between venous congestion and control. Top attributes of venous congestion samples related to regulation of cellular processes (Table 1). We further characterized DEGs of venous congestion by pathway analysis. IPA revealed two of the top associated network functions relating to cell death. Cell death was also reflected in top biological functions along with immunological and inflammatory processes. Similar to arterial ischemia, production of nitric oxide and reactive oxygen species in macrophages was also represented as a top canonical pathway in venous congestion. While IPA for venous congestion also demonstrates immunological and inflammatory processes, cell death and apoptosis appear to be differentiating processes that distinguish venous congestion from arterial ischemia.

### Microarray Validation by qRT-PCR

**Figure 4:**
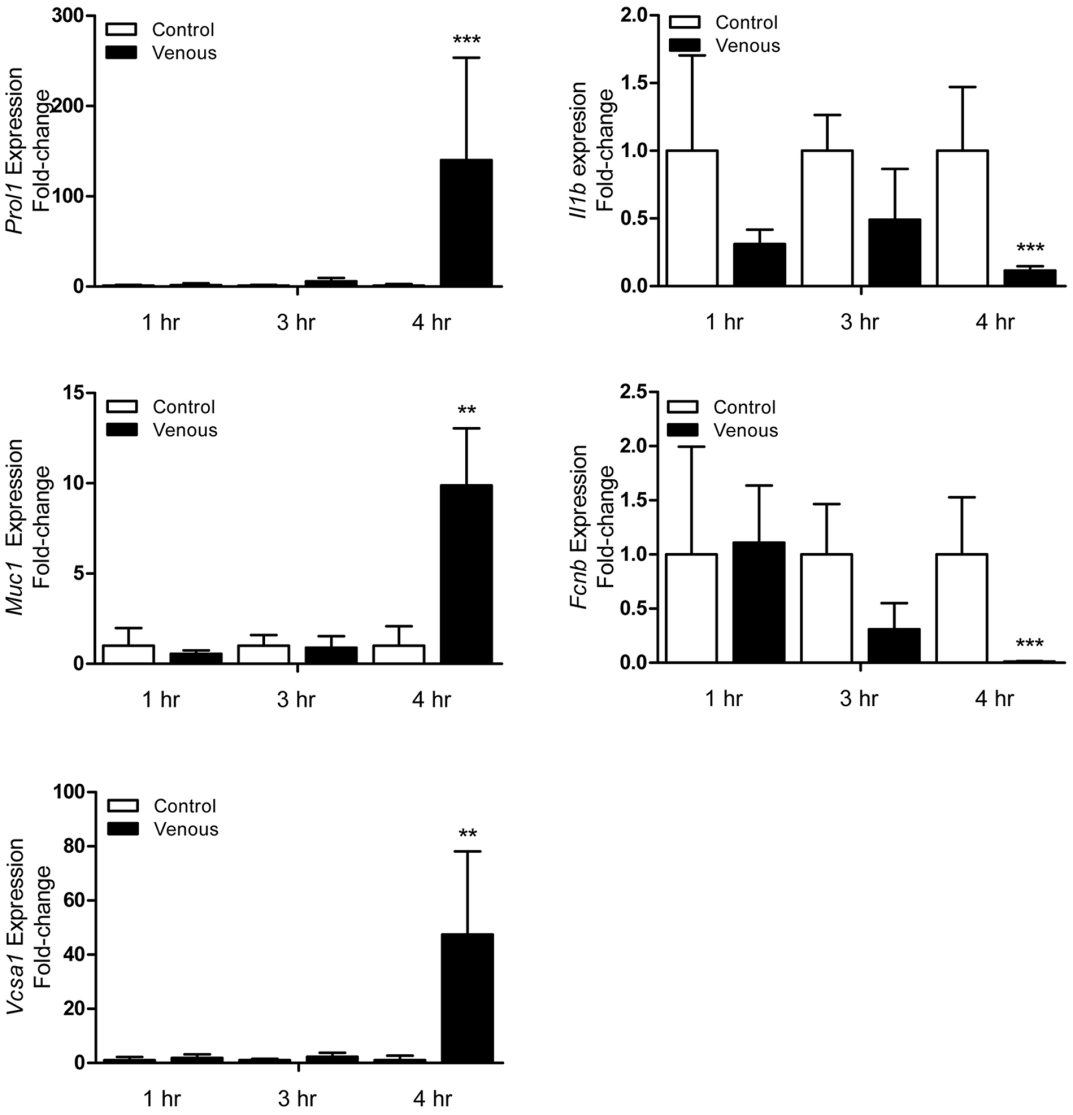
Validation of microarray data using qRT-PCR analysis. qRT-PCR performed on 5 differentially expressed genes (*Prol1, Muc1, Vcsa1, Fcnb, Il1b*) for flaps undergoing both arterial ischemia and venous congestion for 4 hours compared to control. *n = *5 and performed in duplicate. Data are expressed as means ± SE and analyzed by unpaired *t*-test. *P<0.05, **P<0.01, ***P<0.001, cf. control flaps; ^§^ *P<*0.05, cf. arterial ischemia flaps.

**Figure 5:**
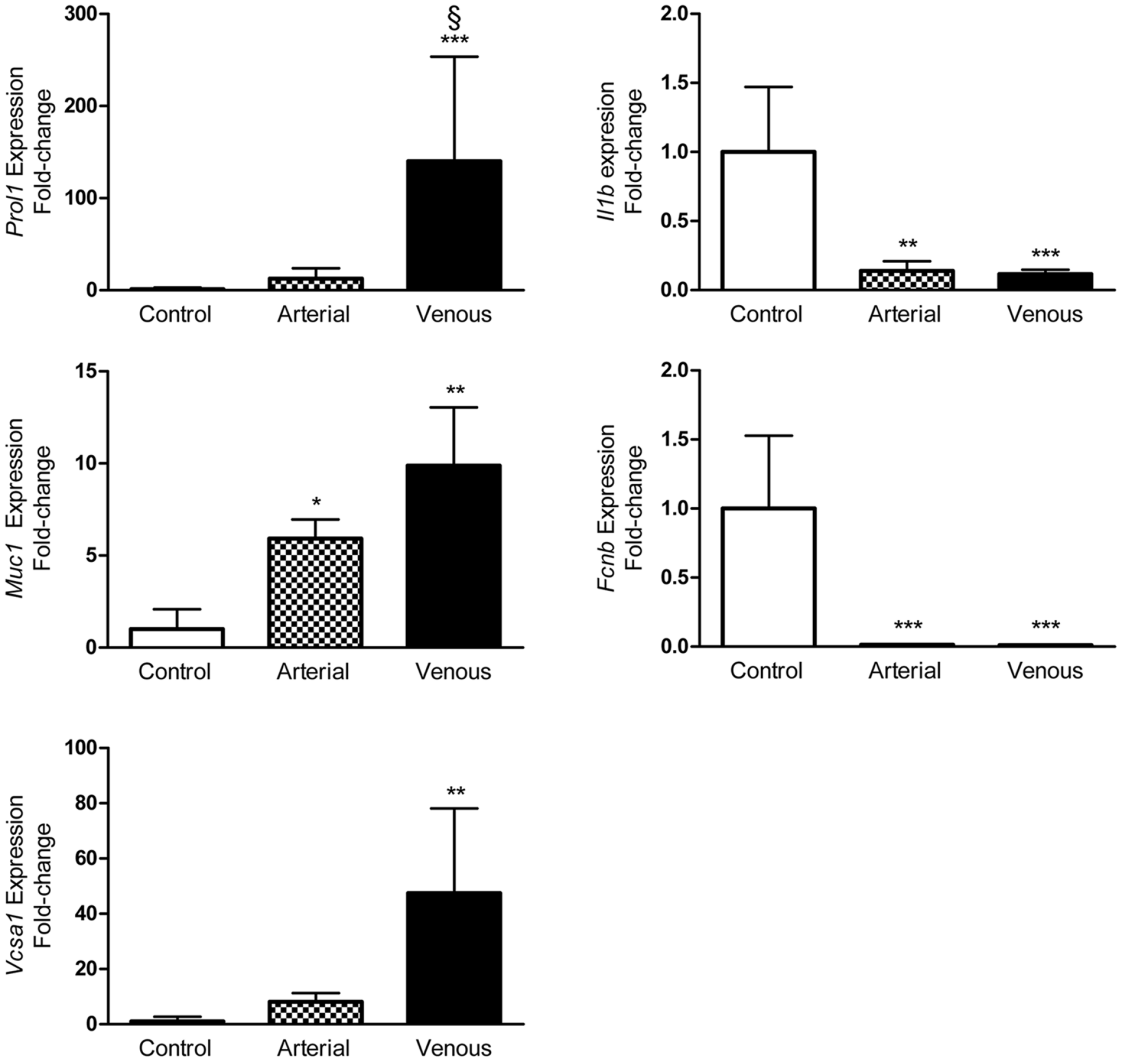
Temporal expression profile of selected genes under venous congestion. qRT-PCR performed on *Prol1, Muc1, Vcsa1, Fcnb, Il1b* for flaps undergoing venous congestion compared to control at *t = *1, 3, 4 hrs. Data are expressed as means ± SE and analyzed by unpaired *t*-test. *P<0.05, **P<0.01, ***P<0.001.

Validation of DEGs in the microarray was performed by qRT-PCR. Because of their magnitude in fold change and relevant pathophysiology, *Prol1*, *Muc1*, *Vcsa1*, *Fcnb*, and *Il1b* expression were analyzed further. qRT-PCR data revealed that *Prol1* expression was not only significantly up-regulated in venous congested flaps compared to control, but was also the only gene validated whose expression was significantly different compared to arterial ischemia flaps (*P = *0.013). The qRT-PCR also confirmed that *Muc1*, *Vcsa1*, and *Prol1* were significantly up-regulated (10-, 47-, and 140-fold, respectively) in flaps undergoing venous congestion. Because the flap is more prone to necrosis when subjected to venous congestion, temporal changes of identified genes were further assessed in samples collected after 1, 3, and 4 hours of venous flap congestion (Figure 4). No significant difference was found in congested flaps when compared to control flaps for 1 and 3 hours across all five genes of interest. Fold change in gene expression of these five markers became much more prominent at 4 hours, which is supported by our earlier experiments in critical ischemia time which lead to irreversible necrosis (Figure 2D). Altogether, the qRT-PCR data confirmed the results of the microarray analysis and suggests that the microarray data as a whole has high validity given our inclusion of multiple biologic replicates (Table 3). The expression of *Prol1 (Muc10), Muc1, Vcsa1, Fcnb,* and *Il1b* was similarly altered as analyzed using both qRT-PCR and microarray (Figure 5 and Table 3).

**Table 3:** Microarray data for genes chosen for validation.

		*Arterial ischemia*		*Venous congestion*	
Gene Symbol (Chr #)	Gene Name	Fold Change	*P* value	Fold Change	*P* value
*Fcnb* (3)	ficolin B	**0.031**	1.12×10^−06^	**0.024**	5.13×10^−07^
*Il1b* (3)	interlukin 1 beta	**0.14**	0.0038	**0.069**	0.00043
*Muc1* (2)	mucin 1, cell surface associated	5.20	0.0029	6.93	0.00093
*Prol1* (14)	proline rich, lacrimal 1	11.77	0.041	110.41	0.0010
*Vcsa1* (14)	variable coding sequence A1	2.98	0.21	33.48	0.0014

## Discussion

Although arterial ischemia and venous congestion have been studied in the literature on various levels, to our knowledge, there has not been a high resolution comparative study of these physiologic phenomenon at the molecular genetic level. Using a well controlled and reproducible rat microvascular flap model, our investigations have highlighted three important principles regarding microvascular flap physiology.

First, arterial ischemia and venous congestion represent two very different pathophysiologic states, as evidenced by their distinct gross, histologic, and transcriptomes. We found that the most differentially expressed genes of venous congestion related to processes involving cell death and apoptosis, whereas gene expression profiles in arterial ischemia relate to immunologic and inflammatory pathways consistent with ischemia-reperfusion injury. Second, although clinical experience suggests that venous congestion may be more immediate and clinically detrimental to flap survival, this head to head study verifies anecdotal experience and provides molecular data that contributes to our mechanistic understanding of flap failure. Our data clearly and consistently demonstrated worse outcomes for flap salvage at all time points of venous congestion relative to similar timepoints in arterial ischemia. Lastly, our experiments have elucidated Prol1 and Vcsa1 as novel candidate biomarkers for venous congestion. The positive repercussions of this finding are likely to manifest in two manners. First, as the most differentially up-regulated gene in venous congestion (140-fold), we anticipate that the identification of Prol1 will lead to further investigations in the exact mechanistic process of venous congestion and will hopefully yield new insights toward its genetic and molecular function.

A more complete mechanistic understanding of the processes of venous congestion and arterial ischemia will play a tremendous role in the development of tissue engineering and bioreactors, facial transplantation, free tissue transfer, and finally composite tissue allograft transplantation of the upper extremities with or without neurally integrated robotics. In order to reliably identify an ischemic flap in danger of necrosis for possible therapeutic intervention, the discovery of early prognostic markers is critical. To our knowledge, there has been only one previous study that examined gene expression profiles in venous occlusion [41]. These authors reported four differentially expressed genes in venous occlusion compared to controls. However the gene expression profile between both arterial ischemia and venous congestion in flaps was not performed. Our genome-wide expression profiling and qRT-PCR analyses demonstrated that there were expression differences to suggest distinct pathophysiological mechanisms of injury. Over 70% (832/1184) of DEGs in venous congestion are distinct from those seen in arterial ischemia. Furthermore, IPA found that flaps subjected to arterial ischemia overwhelmingly demonstrated inflammatory and immune responses, while flaps undergoing venous congestion demonstrated close association with development, function, and cell death of various organ systems. These results provide hard evidence that arterial ischemia and venous congestion operate through distinct pathophysiological pathways.

In our study, we characterized 5 of the most significantly differentially expressed genes in arterial ischemia and venous congestion. Ficolin B (*Fcnb)* was the top down-regulated gene in both arterial ischemia and venous congestion (Table 4). *Fcnb* is one of several ficolins which act as opsonins as part of the innate immune response to aid in phagocytosis by binding pathogens as well as late apoptotic and necrotic cells [49]. Also down-regulated was interleukin-1β (*Il1b),* a well-characterized gene which produces a cytokine involved in a wide array of biological pathways most notably related to the inflammatory response and apoptosis as well as Alzheimer’s disease and type I diabetes mellitus. Of the up-regulated genes, mucin 1(*Muc1)* and *Prol1* (formerly, *Muc10*) are part of the Mucin family of glycoproteins that are known to line the surfaces of epithelial cells. Most notably, overexpression of *Muc1* has been shown to be associated with several types of cancers [50]–[52]. *Prol1* and *Vcsa1* are also opiorphins, an emerging class of peptides that can act as endogenous neutral endopeptidase (NEP) inhibitors [53]. *Vcsa1* encodes for the rat opiorphin homolog sialorphan, known to be responsible for pain suppression, inflammation, and erectile dysfunction [54]. Of note, *Vcsa1* has been found to be one of the most down-regulated genes in three different models of erectile dysfunction in rats [55], [56], and insertion of a *Vcsa1* gene via plasmid vector as well as injection of sialorphin, resulted in restoration of normal erectile function [57]. The authors noted that the restoration of erectile function appeared as result of vasocongestion indicated by the visible edema in the higher doses of plasmids expressing *Vcsa1* compared to control animals. The description of edema and vasocongestion parallels the phenotype of our venous congested flap model. Given that *Vcsa1* and the family of opiorphins also seem to be highly relevant in the pathophysiology of flap failure, the implications of opiorphins in vascular physiology likely extend beyond erectile physiology.

**Table 4:** Microarray data of genes demonstrating highest and lowest fold change.

	Gene Symbol (Chr #)	Gene Name	Fold Change	*P* value
***Top up-regulated genes of venous congestion***	*Prol1* (14)	proline rich, lacrimal 1	110.41	0.0010
	*Vcsa1* (14)	variable coding sequence A1	33.48	0.0014
	*Slc34a2*	solute carrier family 34 (sodium phosphate), member 2	21.21	0.0021
	*Elf5*	E74-like factor 5	14.9	0.0074
	*Rhpn2*	rhophilin, Rho GTPase binding protein 2	14.6	0.0062
***Top down-regulated genes of venous congestion***	*Fcnb* (3)	ficolin B	0.024	5.13×10^−07^
	*Csf3r*	colony stimulating factor 3 receptor (granulocyte)	0.035	2.18×10^−05^
	*Pglyrp1*	peptidoglycan recognition protein 1	0.044	3.93×10^−06^
	*LOC24906*	RoBo-1	0.053	8.12×10^−07^
	*Lilrb4*	leukocyte immunoglobulin-like receptor, subfamily B, member 4	0.055	8.50×10^−05^
***Top up-regulated genes of arterial congestion***	*Sox10*	SRY (sex determining region Y)-box 10	7.39	0.00057
	*Upk1b*	uroplakin 1B	5.40	0.00057
	*Ehf*	ets homologous factor	4.93	0.00075
	*Pcp4*	Purkinje cell protein 4	4.79	7.01×10^−05^
	*Zfp395*	zinc finger protein 395	4.57	0.00086
***Top down-regulated genes of arterial congestion***	*Fcnb (3)*	ficolin B	**0.031**	1.12×10^−06^
	*Csf3r*	colony stimulating factor 3 receptor (granulocyte)	**0.063**	0.000121
	*Pglyrp1*	peptidoglycan recognition protein 1	**0.070**	1.83×10^−05^
	*Mmp8*	matrix metallopeptidase 8	**0.071**	1.02×10^−06^
	*LOC24906*	RoBo-1	**0.074**	2.60×10^−06^

As the most significantly upregulated, venous congestion specific gene (140-fold), Prol1 is an intriguing candidate biomarker. Given the relative paucity of existing published data on Prol1, we are performing further studies to investigate Prol1 function. However, with the identification of Prol1 as a candidate biomarker gene of venous congestion, we hope to steer its discovery toward broader translational clinical applications. We anticipate that Prol1 can be developed as a routine bedside blood test, used for surveillance for early venous congestion in vascularized tissue flaps. We envision that a rapid bedside clinical assay for Prol1 could easily confirm suspected venous congestion; thus, allowing early definitive surgical intervention that could improve free flap salvage. Improved flap salvage rates would minimize patient morbidity and conserve healthcare dollars since microvascular flap reconstructions are among the most complex and expensive procedures performed by reconstructive surgeon. In addition, a venous biomarker may have other clinical applications in fields such as neurosurgery, gastrointestinal surgery, and vascular surgery where detection of early venous compromise would also improve outcomes.

In summary, the data presented above provide insight to the different mechanism through which arterial ischemia and venous congestion may induce deleterious effects to flaps, eventually leading to necrosis and failure. These new insights were achieved through extensive investigation using histopathology and gene expression analysis leading to the discovery of five candidate biomarkers for flap failure. Future experiments looking at altering the expression levels of the five identified candidate genes will reveal the clinical importance as they pertain to successful vascularized flap transplantation.

### Conclusions

We demonstrate for the first time, differences in the gene expression of rat SIEA flaps undergoing arterial ischemia and venous congestion. Further, we have identified a large list of DEGs for the two types of vascular insults. These results offer insight into the mechanistic pathway of flap failure, and may be relevant to the pathogenesis in human flaps. Candidate genes described in this microarray study and validated by qRT-PCR may serve as biological markers for arterial ischemia or venous congestion. *Prol1* in particular demonstrates significant value in differentiating venous congestion from arterial ischemia.

## Supporting Information

Table S1 **Taqman assays provided by Applied Biosystems.**
(DOCX)
