# Supplementary material for: Novel Biomarkers of Arterial and Venous Ischemia in Microvascular Flaps
Source: PLoS One. 2013 Aug 14;8(8):e71628. doi: 10.1371/journal.pone.0071628 (PMC3743756; doi:10.1371/journal.pone.0071628)
Supplement: Table S1 — Taqman assays provided by Applied Biosystems. (DOCX) [file pone.0071628.s001.docx]

**Table S1 Taqman assays provided by Applied Biosystems**

| Assay Name | Taqman Assay ID |
| --- | --- |
| Actb | 4352340E |
| Fcnb (=Fcn1) | Rn00586231_m1 |
| Il1b | Rn00580432_m1 |
| Muc1 | Rn01462585_m1 |
| Muc10 (=Prol1) | Rn00591672_m1 |
| Vcsa1 | Rn01789203_mH |
